# Add-Ons of Heart Disease from the Cardiosurgical Perspective: Gender, Blood Groups and Renal Function

**DOI:** 10.3390/medsci14010158

**Published:** 2026-03-23

**Authors:** Madeline Günther, Dimitrij Zilakov, Ardawan J. Rastan, Sebastian Vogt

**Affiliations:** 1 Heart Surgery, University Hospital Marburg and Giessen, 35043 Marburg, Germany; madeline.guenther@staff.uni-marburg.de (M.G.); a.rastan@uk-gm.de (A.J.R.); 2 Cardiovascular Research Laboratory, Heart Center, Marburg University, 35043 Marburg, Germany; d.zilakov@hotmail.com

**Keywords:** coronary artery disease, valvular heart disease, ABO blood group, sex differences, renal function, retrospective cohort study

## Abstract

Background/Objectives: This retrospective exploratory study aimed to characterize sex-specific patterns of coronary artery disease (CAD) and valvular heart disease (VHD) in a cardiac surgical cohort. In clinical routine, men appear to be more commonly affected by obstructive CAD, whereas women more frequently present valvular heart disease requiring surgical intervention. It remains unclear whether these sex-specific patterns are related to ABO blood groups and selected clinical parameters. Methods: Here, we retrospectively analyzed 983 patients admitted between 2020 and 2024 to a single cardiac centre with CAD and/or VHD requiring valve replacement. Patients were stratified by sex and disease entity (CAD only, CAD + VHD, isolated VHD). ABO and Rhesus factor distributions, cardiovascular risk factors, body mass index (BMI), and renal function (estimated glomerular filtration rate, eGFR) were assessed. Group comparisons were performed using Chi-square and Welch’s *t*-tests. Associations were evaluated using multivariable logistic and linear regression models adjusted for age, BMI, diabetes mellitus, hypertension, smoking, and eGFR. Results: Men were predominantly represented in the CAD-only group, whereas women more frequently underwent valve replacement, either isolated or combined with CAD (*p* < 0.001). When comparing the overall study cohort, blood group O was less prevalent in women than in men (*p* = 0.031), whereas blood group A was more frequent among female patients, although this difference did not reach statistical significance. Moreover, patients with valve disease demonstrated lower eGFR compared with those without valve involvement (men: −6.3 mL/min/1.73 m^2^, *p* = 0.0036; women: −10.4 mL/min/1.73 m^2^, *p* = 0.0019). This effect remained independently associated with reduced eGFR, with women slightly more affected. Conclusions: Gender- specific diseases should be included as secondary diagnoses when considering cardiac surgery. Nephrological complications in the postoperative period can be an important factor in assessing the benefits of surgery. Blood group O was more common in male Patients, suggesting that cardiovascular diseases also exhibit blood group dependence.

## 1. Introduction

Coronary artery disease and valvular heart disease are leading causes of cardiovascular morbidity and mortality and frequently coexist in aging populations [1,2]. Beyond differences in prevalence, accumulating evidence underscores the presence of sex-specific clinical phenotypes. Men typically experience earlier-onset obstructive epicardial coronary artery disease and a higher angiosclerotic burden, whereas women, despite having a similar symptom load, more often exhibit non-obstructive ischaemia, microvascular dysfunction, and distinct risk factor profiles [3,4]. Calcific aortic valve disease likewise demonstrates sex-dependent biology and remodeling [5,6,7]. Women often develop severe aortic stenosis with a lower degree of leaflet calcification and more concentric left-ventricular remodeling [5,8,9]. In this setting, the prognostic impact of concomitant CAD on outcomes after aortic valve intervention differs between men and women [5,6]. Within CAD cohorts, aortic valve sclerosis shows sex-related associations, reflecting shared yet non-identical mechanisms between coronary and valvular disease [9,10]. These observations align with global sex-divergent trends in cardiovascular mortality and highlight persistent gaps in the diagnosis, referral, and treatment of women with cardiovascular disease [4,7,8].

Beyond traditional cardiovascular risk factors such as hypertension, diabetes, dyslipidemia, and smoking, genetic and haematologic determinants have been proposed as potential modulators of disease risk [11]. Among these, the ABO blood group system has drawn particular attention. Non-O blood groups, especially group A, have been associated with a higher risk of CAD [12,13,14,15], thrombotic events [16] and myocardial infarction [17], potentially mediated by elevated circulating levels of von Willebrand factor and factor VIII [18]. However, evidence linking ABO blood groups to valve disease remains scarce and inconsistent, and few studies have examined whether ABO or Rhesus blood groups contribute to the observed sex-specific differences in CAD and VHD [19].

Renal function represents another important determinant of cardiovascular outcomes [20]. Reduced estimated glomerular filtration rate (eGFR) is a well-established predictor of adverse prognosis in CAD [20,21,22]. Less is known, however, about its relationship with valve disease and whether impaired renal function influences sex-specific patterns of disease manifestation [23].

Given these uncertainties, we conducted a retrospective analysis of patients with CAD and/or VHD undergoing cardiac surgery at a single care centre. Our objectives were to (i) characterize sex-specific patterns of CAD and clinically significant VHD requiring surgical intervention, (ii) assess whether ABO and Rhesus blood groups are associated with disease distribution, and (iii) examine the relationship between valve disease and renal function in a sex-stratified manner.

## 2. Materials and Methods

### 2.1. Study Design and Patient Population

This retrospective observational study was conducted at the Department of Cardiac Surgery, University Hospital Marburg (Germany). All consecutive patients admitted between January 2020 and December 2024 who underwent diagnostic evaluation and surgical treatment for coronary artery disease (CAD) or aortic or mitral valve disease were screened. In this study, valvular heart disease (VHD) refers to clinically significant aortic or mitral valve disease requiring surgical valve replacement, either as an isolated indication or in combination with CAD. For readability, the term “valve disease” is used throughout the manuscript to denote this surgically treated VHD cohort. Eligible patients included: (i) isolated CAD confirmed by coronary angiography, (ii) isolated VHD confirmed by echocardiography and/or intraoperative findings, or (iii) combined CAD and VHD.

Patients with other structural heart disease, congenital malformations, or missing ABO blood group data were excluded.

**Figure 1:**
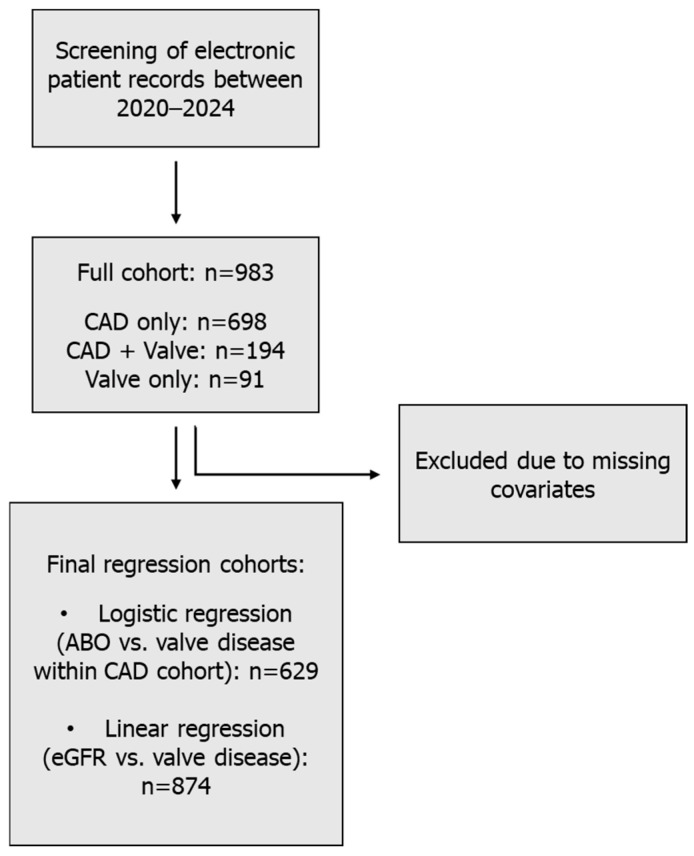
Patient selection flowchart.

The final descriptive cohort comprised 983 consecutive eligible patients, including 698 patients with isolated CAD, 194 patients with CAD and concomitant valve disease, and 91 patients with isolated valve disease (Figure 1).

The final descriptive cohort comprised 983 eligible patients treated between January 2020 and December 2024, including 698 patients with isolated coronary artery disease (CAD), 194 patients with CAD and concomitant valve disease, and 91 patients with isolated valve disease. Numbers of male and female patients are indicated for each subgroup. For regression analyses, patients with incomplete covariate data were excluded using a complete-case approach, resulting in final samples of 629 patients for logistic regression analyses and 874 patients for linear regression analyses.

### 2.2. Data Collection

Demographic data (age, sex, body mass index) and clinical characteristics (hypertension, diabetes mellitus, dyslipidemia, smoking status, renal function) were obtained from electronic health records. ABO blood group and Rhesus factor were determined from routine preoperative laboratory testing. Estimated glomerular filtration rate (eGFR) at admission was calculated from serum creatinine using the 2021 Chronic Kidney Disease Epidemiology Collaboration (CKD-EPI) creatinine equation without a race coefficient, with values indexed to a body surface area of 1.73 m^2^ [24].

### 2.3. Endpoints

The primary endpoint was the distribution of ABO and Rhesus blood groups among patients with CAD, CAD + VHD, and isolated VHD, stratified by sex.

Secondary endpoints included (i) associations between ABO blood groups and valve disease within CAD patients and (ii) associations between valve disease and renal function.

### 2.4. Sample Size and Handling of Missing Data

For regression analyses within the CAD cohort, complete-case analyses were performed for the variables required in the respective multivariable model. Patients with missing values in BMI, eGFR, smoking status, diabetes, or hypertension were excluded from the respective model, and missing covariates were not imputed. This resulted in a final sample size of 629 patients for logistic regression analyses. For analyses of renal function, 874 CAD patients had available eGFR data. Missing covariate data were distributed across all disease subgroups and were not confined to a single subgroup.

Because this was a retrospective exploratory cohort study, no formal a priori power calculation was performed. Data on surgical urgency were not consistently available for all patients and were therefore not included in the present analysis.

### 2.5. Statistical Analysis

Categorical variables are presented as counts and percentages and compared using Chi-square tests or Fisher’s exact test (when expected cell counts were <5). Distributional characteristics of continuous variables were assessed before analysis. Continuous variables are reported as mean ± standard deviation (SD) and compared using Welch’s *t*-test, because of unequal group sizes and potential heterogeneity of variances.

Differences between men and women in disease categories, ABO blood groups, and Rhesus factor were analyzed using Chi-square or Fisher’s exact tests, as appropriate. Model diagnostics were reviewed to assess regression model adequacy, including multicollinearity and, for linear regression models, residual behavior.

Independent associations between ABO blood groups and valve disease were assessed with multivariable logistic regression (reference group: blood group O), adjusted for age, BMI, diabetes, hypertension, smoking, and eGFR. Results are presented as odds ratios (OR) with 95% confidence intervals (CI).

The association between valve disease and renal function was analyzed using multivariable linear regression with eGFR as the dependent variable and valve disease as the predictor, adjusted for age, BMI, diabetes, hypertension, and smoking. Interaction terms (sex × valve) were tested to assess sex-specific effects.

All analyses were two-sided, with *p* < 0.05 considered statistically significant. Analyses were performed using IBM SPSS Statistics, Version 29.0 (IBM Corp., Armonk, NY, USA) and GraphPad Prism version 8.3.0 (GraphPad Software, La Jolla, CA, USA).

## 3. Results

### 3.1. Patients

Baseline characteristics of the final study cohort of 983 patients are summarized in Table 1. Patients were stratified into six groups according to sex and disease status: men with CAD only (*n* = 550), men with CAD and valve disease (*n* = 129), men with isolated valve disease (*n* = 54), women with CAD only (*n* = 148), women with CAD and valve disease (*n* = 65), and women with isolated valve disease (*n* = 37). Overall, this corresponded to 698 patients with isolated CAD, 194 with CAD and concomitant valve disease, and 91 with isolated valve disease. Valve replacement included both aortic and mitral procedures, which were analyzed together. Among men, those with CAD and valve replacement were older than men with CAD only (75.1 ± 9.5 vs. 70.7 ± 10.0 years), while men with valve replacement but no CAD had a similar mean age (70.5 ± 13.1 years). Among women, those with CAD and valve replacement were likewise older than women with CAD only (77.2 ± 9.7 vs. 72.2 ± 10.0 years), whereas women with valve replacement but no CAD had an intermediate mean age (75.8 ± 8.8 years). Mean BMI was similar across all groups (~28 kg/m^2^). The estimated glomerular filtration rate (eGFR) was consistently lower in patients with valve replacement. In men, eGFR was 62.3 ± 21.6 mL/min/1.73 m^2^ in the CAD + valve replacement group, 68.5 ± 22.0 mL/min/1.73 m^2^ in the CAD-only group, and 68.9 ± 20.7 mL/min/1.73 m^2^ in the valve-only group. In women, eGFR was 50.5 ± 20.5 mL/min/1.73 m^2^ in the CAD + valve replacement group, 60.9 ± 23.4 mL/min/1.73 m^2^ in the CAD-only group, and 61.8 ± 24.2 mL/min/1.73 m^2^ in the valve-only group. The prevalence of diabetes, hypertension, and smoking was broadly comparable between groups. Regarding ABO blood groups, distributions were overall similar, although women tended to have a higher proportion of blood group A, whereas men more frequently exhibited blood group O. Rhesus factor distribution did not differ across groups or between sexes.

### 3.2. Distribution of CAD and Valve Disease by Gender

**Figure 2:**
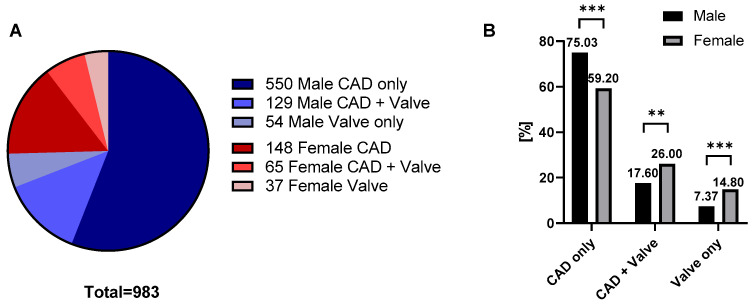
Distribution of CAD and valve replacement in the study population (*n* = 983). (**A**) Overall distribution of patients according to disease category. (**B**) Distribution of disease categories across the male and female cohorts. Woman exhibited a higher prevalence of valve replacement, both with and without concomitant CAD, whereas men were predominantly represented in the CAD-only category (Chi-squared test, *** *p* < 0.001, ** *p* < 0.01).

The pie chart (Figure 2A) illustrates the overall distribution of coronary artery disease and valve disease in the study population. The majority of patients had CAD only, while smaller proportions were diagnosed with CAD in combination with valve disease or with isolated valve disease. When the data were stratified by sex (Figure 2B), marked differences in disease distribution were observed. The CAD-only group was predominantly composed of male patients (74.9% vs. 59.2% in women), whereas women more frequently underwent valve replacement, both in combination with CAD (26.0% vs. 17.6% in men) or as an isolated occurrence (14.8% vs. 7.5% in men). A global Chi-squared test confirmed that the overall distribution of disease categories differed significantly between sexes (*p* < 0.001). In addition, category-specific analyses (Supplementary Table S1) demonstrated significant sex differences within each subgroup: CAD only (*p* < 0.0001), CAD + valve replacement (*p* = 0.0038), and valve replacement only (*p* = 0.0006).

### 3.3. ABO Blood Group Distribution by Gender

**Figure 3:**
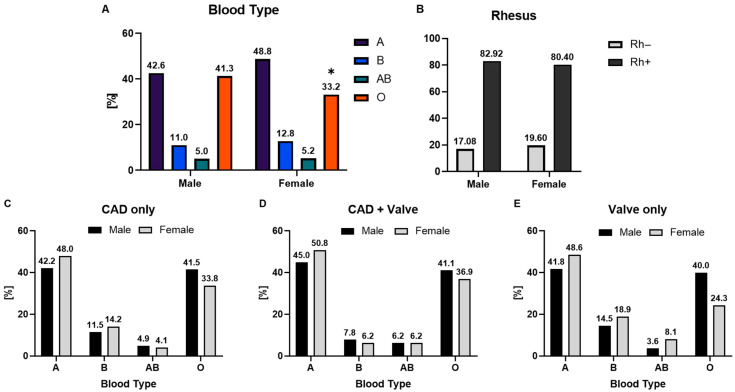
ABO blood group distribution in coronary artery disease and valve disease. (**A**) Overall ABO distribution in CAD and VHD, shown as a summary of the sex-stratified results in panels (**C**–**E**). Bars represent percentages pooled across sexes. Statistical comparison between CAD and VHD cohorts was performed using the chi-square test with * *p* < 0.05. (**B**) Distribution of Rhesus factor (RhD-positive vs. RhD-negative) between men and women within the study population. (**C**–**E**) Sex-stratified ABO distribution: (**C**) CAD only, (**D**) CAD + Valve subgroup, (**E**) VHD only subgroup.

Following the pronounced differences between men and women in terms of disease manifestation, we next examined whether underlying ABO blood group distributions also varied by sex (Figure 3A). In the overall cohort, men were significantly more likely to have blood group O compared with women (41% vs. 33%; *p* = 0.031), whereas no significant differences were observed for groups A, B, or AB, as detailed in the Supplementary Table S2.

To explore whether these sex-specific patterns persisted in clinical subgroups, we analyzed ABO distribution separately in CAD-only, CAD + valve, and valve-only cohorts (Figure 3C–E). In patients with isolated CAD, blood group A was most common (42.2% in men, 48.0% in women), followed by group O (41.5% in men, 33.8% in women; Figure 3C). In the combined CAD and valve subgroup, a similar pattern was observed, with blood group A representing 45.0% of men and 50.8% of women, while group O accounted for 41.1% and 36.9%, respectively (Figure 3D). Among patients with isolated valve disease, blood group A remained the predominant type (41.8% in men, 48.6% in women), followed by group O (40.0% in women, 36.9% in men; Figure 3E). Together, no statistically significant sex differences were detected (all *p* > 0.3), although trends paralleled the overall cohort, with relatively more women being group A and more men group O. Likewise, the distribution of the Rhesus factor did not differ significantly between men and women. Detailed results of the descriptive analyses are summarized in Figure 3.

To evaluate potential associations between ABO blood groups and disease manifestation, multivariable logistic regression analyses were performed in patients with CAD, CAD with concomitant valve disease, and isolated valve disease. Within the CAD cohort (Table 2), comparison of patients with and without valve involvement revealed no significant associations in men, whereas in women, blood group B was independently associated with a lower risk of valve disease compared with blood group O (adjusted OR 0.14, 95% CI 0.02–0.83, *p* = 0.031). No significant associations were observed for blood groups A or AB in either sex.

In a complementary analysis restricted to patients with valve involvement (Supplementary Table S4), CAD + Valve patients were compared with those with isolated valve disease. In this model, none of the ABO groups were significantly associated with isolated valve disease in men. In women, blood group B showed a trend toward higher odds of isolated valve disease compared with CAD + Valve (adjusted OR 5.50, 95% CI 0.72–41.77, *p* = 0.099), although this did not reach statistical significance after adjustment.

### 3.4. Renal Function (eGFR) and Valve Disease

Given the differences in kidney function observed in the baseline characteristics (Table 1), we next examined whether the presence of valve disease was associated with reduced renal function (Table 3). Among CAD patients, those with valve disease had significantly lower estimated glomerular filtration rate compared to those without. In men, the mean difference was −6.3 mL/min/1.73 m^2^ (95% CI −10.5 to −2.1; *p* = 0.0036), and in women −10.4 mL/min/1.73 m^2^ (95% CI −16.8 to −3.9; *p* = 0.0019). Thus, valve disease was consistently associated with impaired renal function in both men and women.

## 4. Discussion

In this retrospective cohort of 983 patients with CAD and/or valvular heart disease, we identified distinct sex-specific patterns of disease distribution. The male population was predominantly affected by CAD without concomitant valve disease, whereas the female population more frequently underwent valve replacement, either in isolation or in combination with CAD. Since only patients with clinically significant VHD requiring intervention were included, our findings specifically reflect patterns in treated valve disease rather than the general VHD population. Beyond these clinical differences, we also observed a significant sex difference in ABO distribution. Women had a lower prevalence of blood group O compared with men, while group A was relatively more common among women. Although this difference did not translate into clear associations with specific disease categories, it aligns with population-based evidence suggesting that non-O groups may be linked to modestly increased cardiovascular risk [13,16,17,18]. Importantly, valve disease was independently associated with impaired renal function, as reflected by consistently lower eGFR values in both sexes.

Our study showed that women in this hospital-based cohort more frequently underwent valve replacement, either alone or in combination with CAD, whereas men were predominantly affected by isolated CAD. This extends previous population-based work, which has consistently demonstrated sex differences in the epidemiology and presentation of aortic stenosis and coronary artery disease [4,6,25,26]. Women with calcific aortic valve disease are known to reach severe stenosis with less valve calcification and typically develop concentric hypertrophy, characterized by thickened ventricular walls and smaller chamber size, whereas men more often are diagnosed with greater valve calcification and eccentric remodeling, marked by chamber dilation and proportionally thinner walls [5,25]. In addition, recent evidence indicates that concomitant CAD influences outcomes after valve replacement differently in men and women [6]. Despite these well-recognized biological and clinical differences, there are still persistent gaps in the referral, diagnosis and treatment of women with cardiovascular disease [8]. By confirming sex-related distributions in a hospital-based cohort, our results provide further evidence that these disparities extend into real-world surgical populations.

In this regard, we examined whether genetic and haematological factors, particularly ABO blood groups, might contribute to the observed patterns of CAD and VHD, given that their role in cardiovascular disease is the subject of ongoing research. Prior studies have identified modest associations between non-O blood types (especially A) and risks of CAD, myocardial infarction, thromboembolism, and dyslipidaemia [12,13,14,18]. However, large cohort analyses such as the Nurses’ Health Study and Health Professionals Follow-up Study, encompassing both women and men, show comparable elevated coronary heart disease risk (~11–23%) without clear sex interaction [27]. Similarly, a population-based donor cohort reported higher thromboembolic risk among non-O groups, but did not analyze sex-specific outcomes [16]. Interestingly, several studies suggest that ABO blood groups may modulate lipid metabolism, specially affecting CAD [27,28,29]; however, they did not analyze whether this was sex-specific. In a French cohort of women, blood group A was independently associated with dyslipidaemia [30]. Since dyslipidaemia is a well-established risk factor for atherosclerosis and may also contribute to valvular calcification, these findings raise the possibility that ABO-related effects on lipid metabolism could influence the balance between CAD and VHD. In our cohort, however, ABO distributions did not significantly differ by disease category, suggesting that such effects, if present, are modest compared with other determinants such as age, renal function, and established cardiovascular risk factors.

Beyond these associations, recent investigations point toward mechanisms more tightly linked to valvular pathology. Although ABO blood groups are known to influence circulating VWF and FVIII levels [31,32], the relevance of this pathway to valve disease remains uncertain in the context of our data. In our cohort, neither ABO nor Rhesus factor was consistently associated with CAD or valve disease overall, suggesting that any potential ABO-related effect is likely small relative to established clinical determinants. Therefore, the VWF/FVIII pathway should be regarded here as a possible biological context rather than a mechanism supported by our study. Future research combining ABO status with biomarker data and more detailed valve phenotyping could help to clarify whether this pathway is clinically relevant in specific subgroups.

Importantly, we also observed that valve disease, irrespective of sex, was independently associated with impaired renal function in both men and women. Renal dysfunction is a well-recognized risk factor for adverse cardiovascular outcomes and has been linked to both CAD and VHD [20,33,34]. Our observation that valve disease was independently associated with lower eGFR, even after adjustment for major risk factors, highlights the importance of the cardio–renal–valvular axis. Chronic kidney disease promotes vascular and valvular calcification through disturbances in mineral metabolism, inflammation, and endothelial dysfunction [20,33]. Large-scale registry data confirm that impaired renal function increases the risk of developing aortic stenosis, with progressively higher risk observed at each stepwise decline in eGFR [35,36]. To our knowledge, few studies have examined this association in a sex-stratified manner. Our findings add to this literature by showing that the relationship between valve disease and impaired renal function is robust across both men and women in our hospital-based cohort.

However, future studies should confirm whether the higher prevalence of valve disease in women is a consistent finding and clarify the mechanisms underlying these differences. Furthermore, this underlines the need for diagnostic strategies that account for sex differences and heightened awareness of atypical disease presentation in cardiac surgery. Finally, the observed association between valve disease and impaired renal function may be clinically relevant, particularly in patients undergoing evaluation for surgical intervention. While this finding is consistent with previous reports on the interplay between renal dysfunction and valvular disease, our data do not allow us to draw conclusions about the underlying mechanisms. Further studies in larger and more phenotypically detailed cohorts are needed to clarify the clinical and pathophysiological significance of this association. Such screening may aid in preoperative risk stratification, perioperative management, and long-term follow-up, particularly in elderly patients and women, who appear to be most affected. Importantly, our findings reflect a surgically treated population with clinically significant disease and should not be extrapolated to patients with milder, medically managed, or inoperable CAD or VHD.

Nevertheless, these findings should be interpreted in light of several limitations. The retrospective single-center design precludes causal inference. In this regard, subgroup analyses for less frequent blood groups (B and AB) were based on small numbers, and the exploratory analyses were not adjusted for multiple comparisons; therefore, subtle associations should be interpreted with caution. Furthermore, men and women were not balanced with respect to baseline characteristics, particularly age; therefore, the observed sex-specific differences should be regarded as descriptive rather than causal. The regression analyses were based on complete cases, as no multiple imputation was performed. In addition, the study population represents a selected cardiac surgical cohort with clinically significant disease requiring intervention, which may limit the generalizability of the results to broader CAD or VHD populations. Finally, aortic and mitral valve disease were analyzed together, and detailed valve phenotyping was not available, limiting disease-specific interpretation.

## 5. Conclusions

In conclusion, this cardiac surgical cohort exhibited sex-specific differences in the distribution of coronary artery disease and clinically significant valvular heart disease requiring surgical intervention. Although exploratory sex-related trends were noted, ABO and Rhesus blood groups were not major determinants of disease distribution. The association between valve disease and reduced renal function requires further investigation in larger, more phenotypically detailed cohorts. These findings should be interpreted as descriptive and exploratory, and may inform future studies on sex-specific patterns of cardiovascular disease in surgical populations.

## Figures and Tables

**Table 1:** Baseline characteristics of the study population stratified by sex and disease category. Patients were categorized into six groups by sex (men, women) and disease status (CAD only, CAD + valve replacement and valve replacement only (without CAD)). Valve replacement included both aortic and mitral valve procedures, which were analyzed together. CAD = coronary artery disease; eGFR = estimated glomerular filtration rate.

Variables	Men CAD Only (*n* = 550)	Men CAD + Valve (*n* = 129)	Men Valve Only (*n* = 54)	Women CAD Only (*n* = 148)	Women CAD + Valve (*n* = 65)	Women Valve Only (*n* = 37)	Overall Cohort (*n* = 983)
Demographics							
Age (yr), mean ± SD	70.7 ± 10.0	75.0 ± 9.5	71.1 ± 12.6	72.2 ± 10.0	77.2 ± 9.7	75.8 ± 8.8	72.1
BMI (kg/m^2^), mean ± SD	28.2 ± 4.5	28.1 ± 3.8	27.8 ± 4.6	28.4 ± 6.0	27.9 ± 4.5	28.4 ± 5.0	28.2
eGFR (mL/min/1.73 m^2^), mean ± SD	68.6 ± 22.0	62.2 ± 21.6	67.7 ± 18.9	60.9 ± 23.4	50.5 ± 20.5	61.8 ± 24.2	65.1
Risk factors							
Diabetes, *n* (%)	207 (37.6%)	46 (35.7%)	10 (18.5%)	57 (38.5%)	22 (33.8%)	10 (27.0%)	357 (36.3%)
Hypertension, *n* (%)	502 (91.3%)	121 (93.8%)	44 (81.5%)	138 (93.2%)	60 (92.3%)	35 (94.6%)	906 (92.2%)
Smoking current, *n* (%)	270 (49.1%)	60 (46.5%)	24 (44.4%)	36 (24.3%)	12 (18.5%)	7 (18.9%)	411 (41.8%)
Blood groups							
O, *n* (%)	228 (41.5%)	53 (41.1%)	22 (40.7%)	50 (33.8%)	24 (36.9%)	9 (24.3%)	386 (39.3%)
A, *n* (%)	232 (42.2%)	58 (45.0%)	22 (40.7%)	71 (48.0%)	33 (50.8%)	18 (48.6%)	434 (44.2%)
B, *n* (%)	63 (11.5%)	10 (7.8%)	8 (14.8%)	21 (14.2%)	4 (6.2%)	7 (18.9%)	113 (11.5%)
AB, *n* (%)	27 (4.9%)	8 (6.2%)	2 (3.7%)	6 (4.1%)	4 (6.2%)	3 (8.1%)	50 (5.1%)
Rhesus							
Rh+, *n* (%)	450 (81.8%)	109 (84.5%)	42 (77.8%)	120 (81.1%)	53 (81.5%)	28 (75.7%)	803 (81.7%)
Rh−, *n* (%)	93 (16.9%)	19 (14.7%)	12 (22.2%)	28 (18.9%)	12 (18.5%)	9 (24.3%)	173 (17.6%)

**Table 2:** Association between ABO blood groups and valve disease in patients with CAD. Logistic regression analyses were conducted within the CAD cohort, comparing patients with concomitant valve disease to those with CAD only. Odds ratios are presented unadjusted and adjusted for age, body mass index, diabetes mellitus, hypertension, smoking status, and estimated glomerular filtration rate. Results are shown separately for men and women, with blood group O as the reference category.

Sex	Blood Group	Unadj. OR (95% CI)	Unadj. *p*	Adj. OR (95% CI)	Adj. *p*	Covariates
Men	A	1.08 (0.71–1.63)	0.731	1.22 (0.74–1.99)	0.437	Age, BMI, DM, Hypertension, Smoking, eGFR
Men	B	0.68 (0.33–1.42)	0.306	0.73 (0.32–1.66)	0.451	Age, BMI, DM, Hypertension, Smoking, eGFR
Men	AB	1.27 (0.55–2.96)	0.573	0.92 (0.32–2.66)	0.878	Age, BMI, DM, Hypertension, Smoking, eGFR
Women	A	0.97 (0.51–1.83)	0.921	1.09 (0.44–2.67)	0.852	Age, BMI, DM, Hypertension, Smoking, eGFR
Women	B	0.40 (0.12–1.28)	0.123	0.14 (0.02–0.83)	0.030	Age, BMI, DM, Hypertension, Smoking, eGFR
Women	AB	1.39 (0.36–5.39)	0.635	1.00 (0.15–6.79)	0.997	Age, BMI, DM, Hypertension, Smoking, eGFR

**Table 3:** Renal function (eGFR) in CAD patients with and without valve disease, stratified by sex. Estimated glomerular filtration rate is presented as mean ± SD for patients with and without concomitant valve disease. Differences in eGFR between groups were assessed using Welch’s *t*-test, with 95% confidence intervals provided. In both men and women, eGFR was significantly lower in patients with valve disease compared to those without.

Sex	No Valve (*n*)	No Valve eGFR (Mean ± SD)	Valve (*n*)	Valve eGFR (Mean ± SD)	Difference (Valve-No) mL/min/1.73 m^2^	95% CI of Difference	Welch *t*	*p*-Value
Men	544	68.5 ± 22.0	128	62.3 ± 21.6	−6.3	−10.5 to −2.1	−2.94	0.0036
Women	140	60.9 ± 23.4	62	50.5 ± 20.5	−10.4	−16.8 to −3.9	−3.17	0.0019
Overall cohort	684	66.9	190	58.4	−8.5	-	-	-

## Data Availability

The original contributions presented in this study are included in the article/Supplementary Materials. Further inquiries can be directed to the corresponding author.

## Supplementary Materials

The following supporting information can be downloaded at: https://www.mdpi.com/article/10.3390/medsci14010158/s1, Table S1: Category-specific Chi-square analyses of sex differences in disease distribution; Table S2: Sex-specific ABO distribution of the whole study population; Table S3: Sex-specific distribution of ABO blood groups in patients with CAD and valve disease; Table S4: Logistic regression analysis between ABO blood groups and isolated valve disease compared with CAD + Valve (sex-stratified).
