# Supplementary material for: Add-Ons of Heart Disease from the Cardiosurgical Perspective: Gender, Blood Groups and Renal Function
Source: Med Sci (Basel). 2026 Mar 23;14(1):158. doi: 10.3390/medsci14010158 (PMC13028197; doi:10.3390/medsci14010158)
Supplement: Supplementary file 1 [file medsci-14-00158-s001.zip › medsci-4137671-supplementary.pdf]

## Supplementary Materials

*Table S1. Category-specific Chi-square analyses of sex differences in disease distribution*

Category-specific Chi-square analyses of sex differences in disease distribution. The table shows the results of 2 × 2 Chi² tests comparing men and women within each disease category (CAD only, CAD + valve replacement, valve replacement only). Significant differences were observed across all categories. Valve replacement includes both aortic and mitral procedures. CAD = coronary artery disease.

**Table S1.**

| Cohort | Men (n) | Women (n) | p-value |
|---|---|---|---|
| CAD only | 550 | 148 | **<0.0001** |
| CAD + Valve | 129 | 65 | **0.0038** |
| Valve only | 54 | 37 | **0.0006** |

*Table S2. Sex-specific ABO distribution. P-values from Chi-square tests with Yates' correction; two-sided.*

**Table S2.**

| Blood Group | Men (n) | Women (n) | Total (n) | % Overall (n=983) | p-value |
|---|---|---|---|---|---|
| A | 312 | 122 | 434 | **44.2%** | 0.115 |
| B | 81 | 32 | 113 | 11.5% | 0.513 |
| AB | 37 | 13 | 50 | 5.9% | 1.000 |
| O | 303 | 83 | 386 | 39.3% | **0.031** |

*Table S3. Sex-specific distribution of ABO blood groups in patients with CAD and valve disease*

Sex-specific distribution of ABO blood groups in patients with CAD and valve disease. The table presents the distribution of ABO blood groups (A, B, AB, O) stratified by sex within three diagnostic categories: isolated CAD, CAD with concomitant valve disease, and isolated valve disease. Values are given as absolute numbers with percentages in parentheses. P-values refer to comparisons between men and women within each subgroup (chi-square or Fisher's exact test, as appropriate).

**Table S3.**

| | Blood group | Men, n (%) | Women, n (%) | p-value | Overall n (%) |
|---|---|---|---|---|---|
| CAD only | O | 228 (39.0) | 50 (29.1) | 0.14 | 278 (37.4) |
| | A | 232 (39.7) | 71 (41.3) | 0.20 | 303 (40.8) |
| | B | 63 (10.8) | 21 (12.2) | 0.41 | 84 (11.3) |
| | AB | 27 (4.6) | 6 (3.5) | 0.85 | 33 (4.4) |
| Total | | 550 | 148 | | 698 |
| CAD + Valve | O | 53 (41.1) | 24 (36.9) | 0.66 | 77 (39.5) |
| | A | 58 (45.0) | 33 (50.8) | 0.57 | 91 (46.7) |
| | B | 10 (7.8) | 4 (6.2) | 0.90 | 14 (7.2) |
| | AB | 8 (6.2) | 4 (6.2) | 1.00 | 12 (6.2) |
| | | 129 | 65 | | 194 |
| Valve only | O | 22 (33.3) | 9 (23.7) | 0.14 | 31 (33.7) |
| | A | 22 (33.3) | 18 (47.4) | 0.68 | 41 (44.6) |
| | B | 8 (12.1) | 7 (18.4) | 0.86 | 15 (16.3) |
| | AB | 2 (3.0) | 3 (7.9) | 0.68 | 5 (5.4) |
| Total | | 55 | 37 | | 92 |

*Table S4. Logistic regression analysis between ABO blood groups and isolated valve disease compared with CAD+Valve (sex-stratified)*

Association between ABO blood groups and isolated valve disease compared with CAD+Valve (sex-stratified). Logistic regression analyses were performed with CAD+Valve as the reference group and valve only as the outcome. Odds ratios (OR) are presented unadjusted and after adjustment for age, body mass index (BMI), diabetes mellitus, hypertension, smoking status, and estimated glomerular filtration rate

(eGFR). Results are shown separately for men and women, with blood group O as the reference category.

**Table S4**

| **Sex** | **Blood Group** | **Valve risk (%)** | **Unadj OR (95%CI)** | **Unadj p** | **Adj OR (95%CI)** | **Adj p** | **Covariates** |
|---|---|---|---|---|---|---|---|
| Men | A | 32.9% | 0.92 (0.44-1.91) | 0.817 | 0.95 (0.42-2.13) | 0.901 | Age, BMI, DM, Hypertension, Smoking, eGFR |
| Men | B | 32.9% | 1.56 (0.51-4.79) | 0.441 | 2.07 (0.61-7.02) | 0.241 | Age, BMI, DM, Hypertension, Smoking, eGFR |
| Men | AB | 32.9% | 0.40 (0.04-3.66) | 0.417 | 0.56 (0.06-5.53) | 0.623 | Age, BMI, DM, Hypertension, Smoking, eGFR |
| Women | A | 41.8% | 1.45 (0.51-4.10) | 0.489 | 1.58 (0.52-4.78) | 0.419 | Age, BMI, DM, Hypertension, Smoking, eGFR |
| Women | B | 41.8% | 6.38 (1.05-38.86) | 0.045 | 5.50 (0.72-41.77) | 0.099 | Age, BMI, DM, Hypertension, Smoking, eGFR |
| Women | AB | 41.8% | 2.12 (0.25-17.93) | 0.488 | 1.64 (0.16-16.79) | 0.679 | Age, BMI, DM, Hypertension, Smoking, eGFR |
